# Atypical Presentation of Syphilis With a Superimposed Methicillin-Resistant Staphylococcus aureus Infection

**DOI:** 10.7759/cureus.86133

**Published:** 2025-06-16

**Authors:** Kaitlyn Novotny, Claire V Ong, Michael V Dicaro, Aditi Singh

**Affiliations:** 1 Internal Medicine, Kirk Kerkorian School of Medicine at the University of Nevada, Las Vegas, Las Vegas, USA

**Keywords:** homeless, methicillin-resistant staphylococcus aureus, mrsa, substance use, syphilis

## Abstract

Syphilis, caused by the organism *Treponema pallidum*, is a sexually transmitted infection that typically presents with a painless ulcer on the genitalia or oral mucosa. Primary syphilitic ulcers can go unnoticed and progress to more serious presentations, including disseminated cutaneous disease, neurologic disease, and even death. Early recognition and treatment can resolve the infection. Some infections are complicated by abnormal presentations or masked by additional infections. Risk factors for presentation variances include undomiciled persons or people who regularly participate in high-risk sexual activities. We present a case of disseminated syphilis with superimposed superficial methicillin-resistant *Staphylococcus aureus *infection in an immunocompetent patient with a history of homelessness and IV drug use.

## Introduction

Syphilis, caused by the bacterium *Treponema pallidum*, is a sexually transmitted infection (STI) with a well-defined progression through primary, secondary, and tertiary stages. Primary syphilis typically presents as a painless, erythematous ulcer involving the genitalia, anus, or oral mucosa. If left untreated, the infection may advance to secondary syphilis, characterized by systemic symptoms including rashes, lymphadenopathy, and mucosal lesions. In tertiary syphilis, the disease involves multiple organ systems, including the cardiovascular and nervous systems, potentially leading to life-threatening complications [1]. Syphilis remains a significant public health issue, especially among vulnerable populations such as the unhoused, immunocompromised, and those participating in high-risk sexual activity [2]. One study determined that 6498 people between 1968 and 2015 died from syphilis-related diseases and reported increasing rates of congenital syphilis directly correlating to increasing rates of adult infections [3]. Prompt diagnosis and treatment are essential to prevent disease progression and transmission. 

Syphilis can also present atypically. Atypical manifestations include painful chancres, chancres localized in sites other than the anogenital area, erosions, erythema, edema, balanitis, and simultaneous presentation of more than a single stage of the disease [1,4]. The possible co-occurrence of other infections in high-risk individuals adds an additional layer of complexity, especially in low-resource settings. Methicillin-resistant *Staphylococcus aureus* (MRSA), a relatively common cause of soft tissue and skin infections, can present as superficial skin lesions or localized abscesses. Purulent lesions are frequently seen in cases of MRSA infection. When these two infections overlap, they can obscure the clinical picture and complicate diagnosis and treatment. In this presentation, we report on the peculiar presentation of a syphilis with superimposed MRSA infection in a middle-aged male patient.

## Case presentation

**Figure 1:**
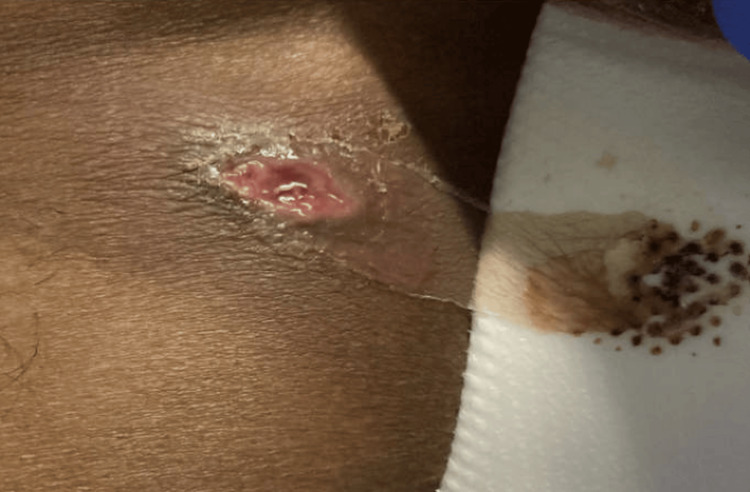
Wound over the left anterior iliac spine.

**Figure 2:**
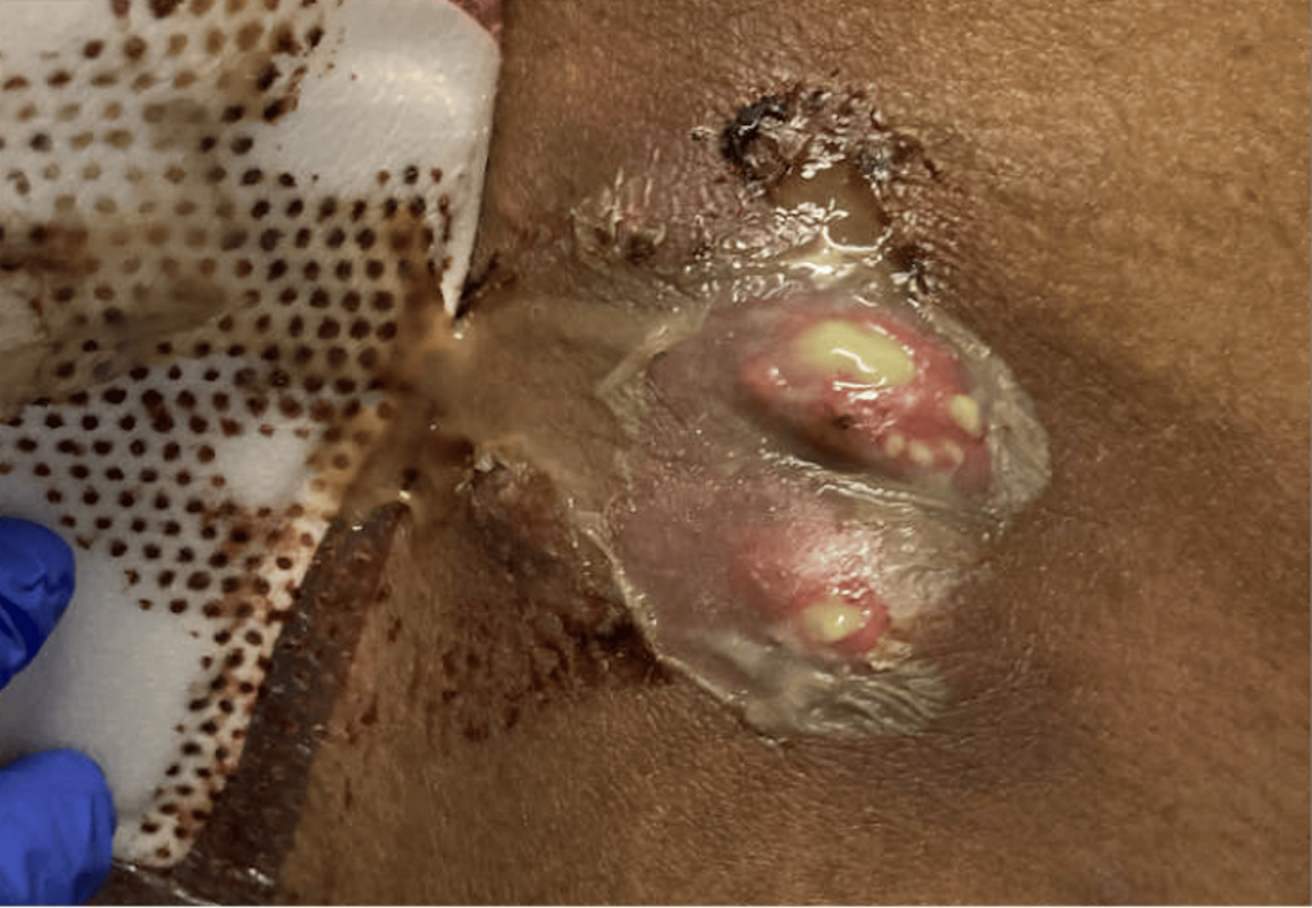
Wound over the right anterior iliac spine.

**Figure 3:**
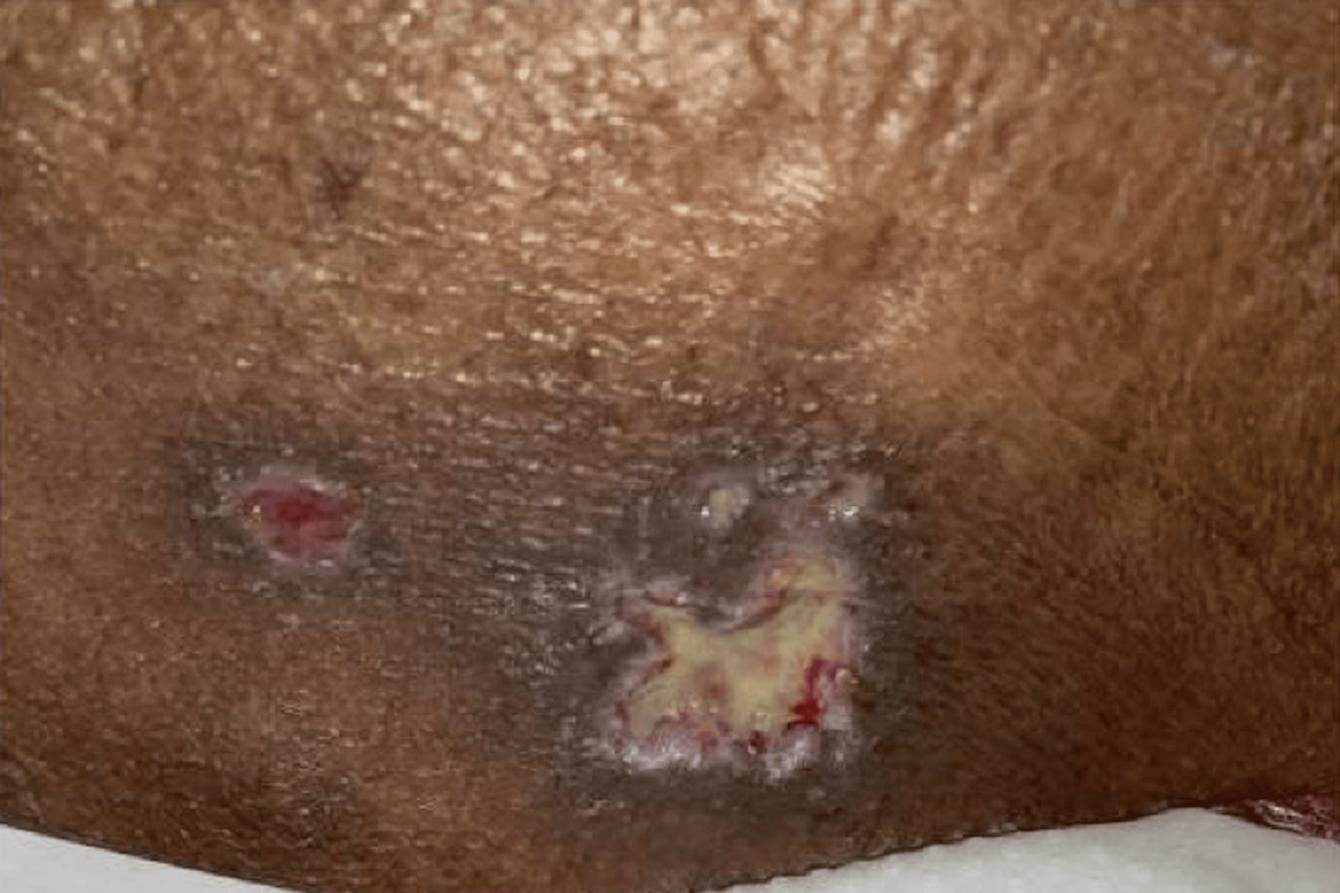
Wound on the left lateral hip.

A 38-year-old man with a history of methamphetamine misuse presented to the emergency department with a chief complaint of tender right-sided inguinal lymphadenopathy. In addition to his lymphadenopathy, he also had multiple purulent ulcers over his anterior iliac spines bilaterally, as seen in Figure 1 and Figure 2, and a 2x1.5x1.5 cm deep, purulent wound on the lateral left hip superficial to his femoral head (Figure 3). The patient also presented with several smaller, purulent lesions on his lower extremities, abdomen, and pelvis.

During evaluation, the patient expressed that he first noticed his lymphadenopathy a week prior to admission, when he awoke to find his left hip swollen and erythematous. As the week progressed, the lesion expanded and became purulent (Figure 3), and additional wounds appeared in the aforementioned anatomical locations. Further investigation revealed that the patient had engaged in unprotected sex with a sex worker approximately a month before admission.

A review of the patient’s symptoms revealed no significant findings; the patient denied fever, chills, fatigue, sore throat, muscle pain, or unintended weight loss during the previous two months. The patient's vitals were stable and non-revealing. During his stay in the hospital, the patient remained afebrile. Aside from the lymphadenopathy and purulent wounds, his physical examination was unremarkable. No other skin changes, rashes, visual or auditory symptoms, or neurologic or motor deficits were appreciated or reported.

Given this patient’s sexual history and presenting symptoms, various laboratory tests were ordered, including a complete blood count (CBC), treponemal syphilis IgG antibody test, and rapid plasma reagin (RPR). The CBC revealed an elevated white blood cell (WBC) count of 19,000 µL (normal WBC: 4,000-11,000 µL). The treponemal syphilis IgG antibody test was positive, and his RPR was also positive with the RPR titer elevated at 1:64 (normal titer <1:2). The patient denied any history of prior syphilis infection or treatment, and there were no labs in his chart indicating an infection in the past. Therefore, we believe that these results indicate a recent syphilis infection. Further testing for other STIs and diseases, such as HIV, hepatitis, gonorrhea, and chlamydia, was negative. Blood cultures were negative.

**Figure 4:**
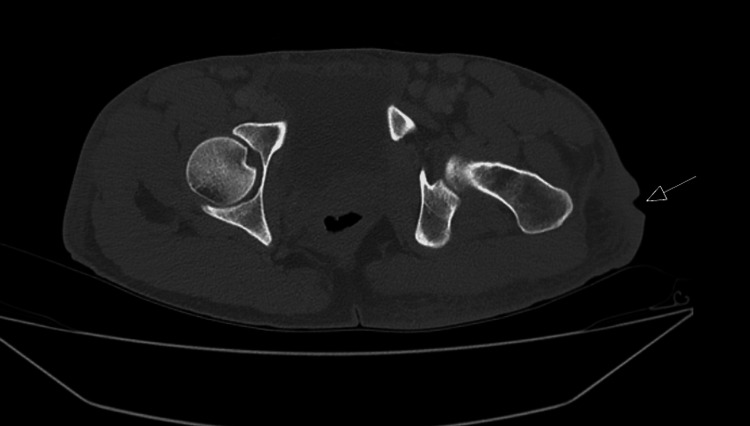
CT scan of the abdomen and pelvis showing superficial infection of the left lateral hip without osteomyelitis. The arrow indicates the area of infection.

The positive syphilis antibody test, while significant, did not explain the patient's diffuse, painful, purulent ulcers, which prompted further testing. A culture of the primary wound on the patient's left, lateral hip grew MRSA and also tested positive for the MRSA-associated PBP2a protein. Due to concerns for deep tissue infections such as osteomyelitis or abscesses, a computed tomography (CT) abdomen and pelvis with contrast was performed, which was unremarkable (Figure 4).

The patient was started on vancomycin and cefepime for MRSA and gram-negative coverage. He was also given intramuscular penicillin G to treat syphilis. Infectious disease specialists were consulted, and the antibiotic regimen was adjusted to vancomycin and cefepime. The patient’s MRSA treatment was further simplified to a single dose of dalbavancin. He was advised to complete two additional doses of intramuscular penicillin G over the following weeks to ensure eradication of syphilis.

The patient showed significant clinical improvement after several days of antibiotic therapy. After a one-week hospital stay, the patient was discharged with a plan for close outpatient follow-up to complete his treatment for syphilis and to monitor for any recurrence of MRSA.

## Discussion

While syphilis usually follows a well-characterized, progressive course through its primary, secondary, and tertiary stages, atypical manifestations of the infection can occur, posing diagnostic and therapeutic challenges. Moreover, co-infection with syphilis and other organisms causing skin or soft tissue infections may obscure the clinical picture, creating a diagnostic and therapeutic conundrum.

Primary syphilis typically presents with a painless chancre on the anogenital region or within the oropharynx [5]. Secondary syphilis occurs after a primary infection in which circulating immune complexes deposit into organs and tissues, causing systemic and cutaneous signs and symptoms [6]. Secondary syphilis typically occurs 6-12 weeks after initial infection but can present as far out as six months after inoculation [7]. Tertiary syphilis varies in its presentation and is typically separated into three categories: neurological, cardiovascular, or gummatous [5,8]. Some patients experience latent syphilis, which is an asymptomatic manifestation of a syphilis infection [5]. Latent syphilis typically follows secondary syphilis and precedes the development of tertiary syphilis [8]. Latent syphilis can revert to secondary syphilitic presentations early on or progress to the more serious presentation of tertiary syphilis [8]. Numerous atypical presentations of syphilis have been described, and as discussed previously, include painful chancres, chancres occurring outside the anogenital region, and cases where multiple stages of the disease manifest simultaneously [1,4]. Other forms include psoriasiform secondary syphilis, which presents with plaque-like lesions that resemble psoriasis plaques and an eczematous-type syphilis manifestation [4]. These are just a few irregular ways syphilis can present, making its diagnosis difficult and earning it the nickname “the great imitator.”

Cutaneous MRSA infections, like syphilis, exhibit a wide spectrum of clinical manifestations, ranging from subclinical inoculation to life-threatening systemic involvement. These infections may present as localized lesions, most commonly abscesses, or as more extensive involvement of the skin layers and larger body areas, as seen in cellulitis. Patients often present with a combination of fluctuant abscesses and accompanying cellulitis [9]. Those who are immunocompromised or suffering from other illnesses may experience more aggressive forms of the disease course or atypical manifestations. Cases of atypical cutaneous MRSA infections have been described in which the infection resembles the rash of the varicella zoster virus [10].

This case demonstrates an irregular manifestation of secondary syphilis in a patient who denied a history of painless anogenital or oral ulcers, further complicating his final diagnosis. He had no knowledge or evidence of primary infection, and his cutaneous wounds were purulent, painful, and occurred outside of the anogenital region, where syphilis usually presents. The lymphadenopathy was painful, when, clinically, most patients with syphilis experience painless lymphadenopathy. On exam, there was no evidence of the well-described maculopapular rash of secondary syphilis nor features of tertiary syphilis. Thus, this presentation was more consistent with a generalized skin infection, so his primary wound was cultured, revealing MRSA. Infection with this bacterium explained the ulcers' purulent and painful characteristics as well as the painful lymphadenopathy. Given his negative HIV tests, the infection's dissemination was likely due to the underlying syphilis infection, whose presence was masked by the superimposed MRSA infection and incidentally confirmed with an RPR titer due to his disclosure of high-risk sexual activity. The patient was recommended to follow up in the clinic after discharge from the ED, where additional workup may be done as indicated. The presence of both infections made this patient's diagnosis difficult, as his symptoms could not be attributed to a singular infection and, therefore, required a broader differential.

The purpose of this discussion is to highlight the need for maintaining a low threshold for diagnostic investigation in patients who are at high risk of various infections. Ultimately, this patient engaged in multiple practices that made him at high risk for infection with both syphilis and MRSA. At the time of presentation, he admitted to methamphetamine abuse and high-risk sexual behaviors, and endorsed that he was undomiciled. Studies show that those who engage in high-risk sexual activity, such as this patient, are at an increased risk of acquiring an MRSA infection [11]. Another article studying the association between *S. aureus* colonization and HIV infections found that *S. aureus *was not directly associated with HIV but was instead associated with illegal substance use [12]. The same study found a heightened risk of *S. aureus* colonization, particularly in the genital and adjacent areas, among individuals recently infected with *Treponema pallidum *[12]. Other studies have shown that the rate of syphilis infections in the homeless population is higher than the average rate of non-homeless adults [2]. These findings support the observations in this case report, as this patient not only had an increased risk of *Staphylococcus aureus* infection from his methamphetamine use but was also at risk due to his unknown syphilis infection, which he was at a higher risk of acquiring due to his housing situation. Similar case studies have reported on MRSA infections in men who participate in high-risk sexual behaviors. One such study describes an abnormal presentation of an MRSA infection causing genital ulceration as well as lymphadenopathy in their patient [13]. Though similar to this report's case study, the ulcerative lesion on this patient’s swollen lymph nodes, in addition to the positive RPR, complicated the diagnosis of both syphilis and MRSA, thus highlighting the importance of thorough history-taking and consideration of patient risk factors when creating a differential for complex infectious processes.

While *S. aureus* colonization is commonly associated with unsanitary living conditions, illicit substance abuse, and STIs such as syphilis, few case reports describe the unique presentation of an MRSA infection superimposed on a syphilis infection. This case presents a distinctive diagnostic and therapeutic challenge involving an initially positive RPR titer in the absence of the typical signs of syphilis. This case underscores the perplexing presentation faced by the patient and serves as a reminder for physicians to maintain a broad differential diagnosis when managing atypical infectious presentations. Additionally, it emphasizes the importance of avoiding diagnostic anchoring based on an isolated positive test result and taking full patient histories to assess their risks and behaviors. In doing so, clinicians can correctly focus on differentials when presented with a complex or atypical presentation of some infectious process.

## Conclusions

In conclusion, our case describes an abnormal presentation of secondary syphilis. Rather than presenting with a typical maculopapular appearance, we saw several ulcerative and purulent lesions in this patient's perigenital region and dispersed across his lower extremities. This confusing clinical picture was due to a superimposed MRSA infection that ultimately overshadowed his syphilitic symptoms and challenged our differentials. As few case reports have been published discussing such an abnormal presentation, we hope our findings will help guide future practitioners in assisting with diagnosis and treatment.
